# Dietary fibre and metabolic health: A clinical primer

**DOI:** 10.1002/ctm2.70018

**Published:** 2024-10-13

**Authors:** Valentin Mocanu, Karen L Madsen

**Affiliations:** ^1^ Digestive Disease and Surgery Institute Cleveland Clinic Ohio USA; ^2^ Department of Medicine University of Alberta Edmonton Alberta Canada

**Keywords:** fibre, metabolism

## Abstract

Dietary fibres consist of a heterogeneous group of carbohydrate polymers which resist digestion by human gastrointestinal enzymes. Consumption of dietary fibre has been linked with innumerable health benefits encompassing the foundational pillars of metabolic health from obesity to hypertension, dyslipidaemia, and type 2 diabetes mellitus (T2DM), with many of these benefits linked with metabolites produced by the fermentation of fibre by gut microbes [1]. The potential of dietary fibre to provide a safe and effective nonpharmacologic complementary tool with which to combat the evolving consequences of the obesity epidemic has recently garnered tremendous attention in both medical literature and lay media alike.

**Highlights:**

1. Consumption of dietary fibre has been linked with innumerable health benefits encompassing the foundational pillars of metabolic health with many of these benefits linked with metabolites produced by the fermentation of fibre by gut microbes
2. At the present time clinicians are faced with an impossible task in which there is rapidly mounting evidence for dietary fibres advancing metabolic health, but little practical options for healthcare providers other than to simply recommend patients consume more fibre.
3. Benefits of fibre intake may perhaps be maximised in an individual by matching specific fibre consumption with existing microbial functional characteristics
4. If dietary fibres could be demonstrated to act as successful adjuncts to sustain or improve standard of care therapies or even alleviate common gastrointestinal side effects associated with current treatments, they would be an invaluable tool in our metabolic health armamentarium. Neither Dr. Madsen or I have any financial conflicts of interest pertinent to the contents of this manuscript.

## 1 INTRODUCTION

Dietary fibres consist of a heterogeneous group of carbohydrate polymers which resist digestion by human gastrointestinal enzymes. Consumption of dietary fibre has been linked with innumerable health benefits encompassing the foundational pillars of metabolic health from obesity to hypertension, dyslipidaemia, and type 2 diabetes mellitus (T2DM), with many of these benefits linked with metabolites produced by the fermentation of fibre by gut microbes.^1^ The potential of dietary fibre to provide a safe and effective nonpharmacologic complementary tool with which to combat the evolving consequences of the obesity epidemic has recently garnered tremendous attention in both medical literature and lay media alike.

### 1.1 Fibre intake for prevention of metabolic disease

There is little debate amongst experts regarding the preventative value of dietary fibre consumption with regards to the complete spectrum of metabolic health. Landmark work by Ludwig et al. in the late 1990s provided perhaps the first large‐scale epidemiological correlation between dietary fibre and cardiovascular disease risk prevention.^2^ Using the CARDIA multicentre US cohort study over a ten‐year follow‐up of 2909 healthy individuals, the authors demonstrated that fibre consumption strongly predicted reductions in cardiovascular risks like weight gain, insulin levels, hypertension, and cholesterol.^2^ This initial study spurred intense population‐level research into the effect of fibre on prevention of incidence of metabolic disease, culminating in overwhelming support for its use as a preventative tool for cardiovascular and metabolic health. Numerous high‐quality systematic reviews and meta‐analyses have since provided conclusive evidence for the use of dietary fibre in prevention of all aspects of metabolic disease.^3^, ^4^

However, despite the numerous purported health benefits associated with dietary fibre, a dramatic gap in fibre consumption exists with less than 5% of all Americans thought to consume their recommended daily fibre intake (25 g per day for females and 38 g per day for males) as energy‐dense highly processed foods have taken the place of high fibre foods.^5^ In addition, questions that have yet to be answered include identifying in which form it is best to consume fibres to maximise metabolic health. Limited research has directly compared the efficacy of dietary versus supplemental fibres. Fibres found in whole foods are associated with a host of micro‐ and macronutrients, themselves associated with multiple metabolic and nonmetabolic health benefits making it challenging to discern benefits solely attributed to fibre intake.

The development of isolated and synthetic fibres provide avenues for targeted therapy of metabolic disorders as different combinations of fibres could be rationally selected based on their unique physiochemical properties, which differentially affect gut microbial fermentation and subsequent effects on host physiology.^1^, ^6^ However, not all fibre supplements exert metabolic benefit in all individuals; indeed, some types of fibre, such as β‐fructan fibres found in foods such as artichoke, chicory roots, garlic, asparagus, and bananas have the potential to induce inflammation if the microbes needed to ferment the fibre are not present in the individual and the unfermented fibre reaches the colon.^7^

### 1.2 Fibre intake as therapy for metabolic diseases

In contrast to the well‐accepted role of dietary fibre as a preventative tool for metabolic health, its use as therapy for established disease remains controversial. It is unclear why the convincing population‐level benefits fail to consistently demonstrate durable real‐world efficacy through interventional trials involving fibre. One possibility is that confounders at a population level cannot be fully accounted for leading to a variety of individual or societal factors which may instead be responsible for explaining the observed effects.

When considering the pillars of metabolic health, the greatest controversy surrounds the role of fibre as a therapeutic modality for obesity. Numerous well conducted systematic reviews indeed demonstrate body weight improvements with short‐term fibre supplementation independent of caloric restriction. In a meta‐analysis of 62 randomised trials comparing fibre supplementation to comparator diets, dietary fibres were found to significantly reduce mean body weight. These effects, however, were modest with a mean body weight loss of 0.33 kg and no overall change in body fat percentage.^8^ While statistically significant, these findings highlight the relative ineffectiveness of using fibre as a stand‐alone therapy as the effects pale in comparison to those of novel weight loss medications like enteroendocrine agonists. However, in contrast to obesity, the effects of interventional fibre on the other pillars of metabolic health such as dyslipidaemia, hypertension and T2DM appear more robust. Evidence from systematic reviews and meta‐analyses demonstrate consistent improvements in measurements of blood pressure, serum cholesterol, and glycaemic markers with enhanced fibre intake.^9^, ^10^

**FIGURE 1:**
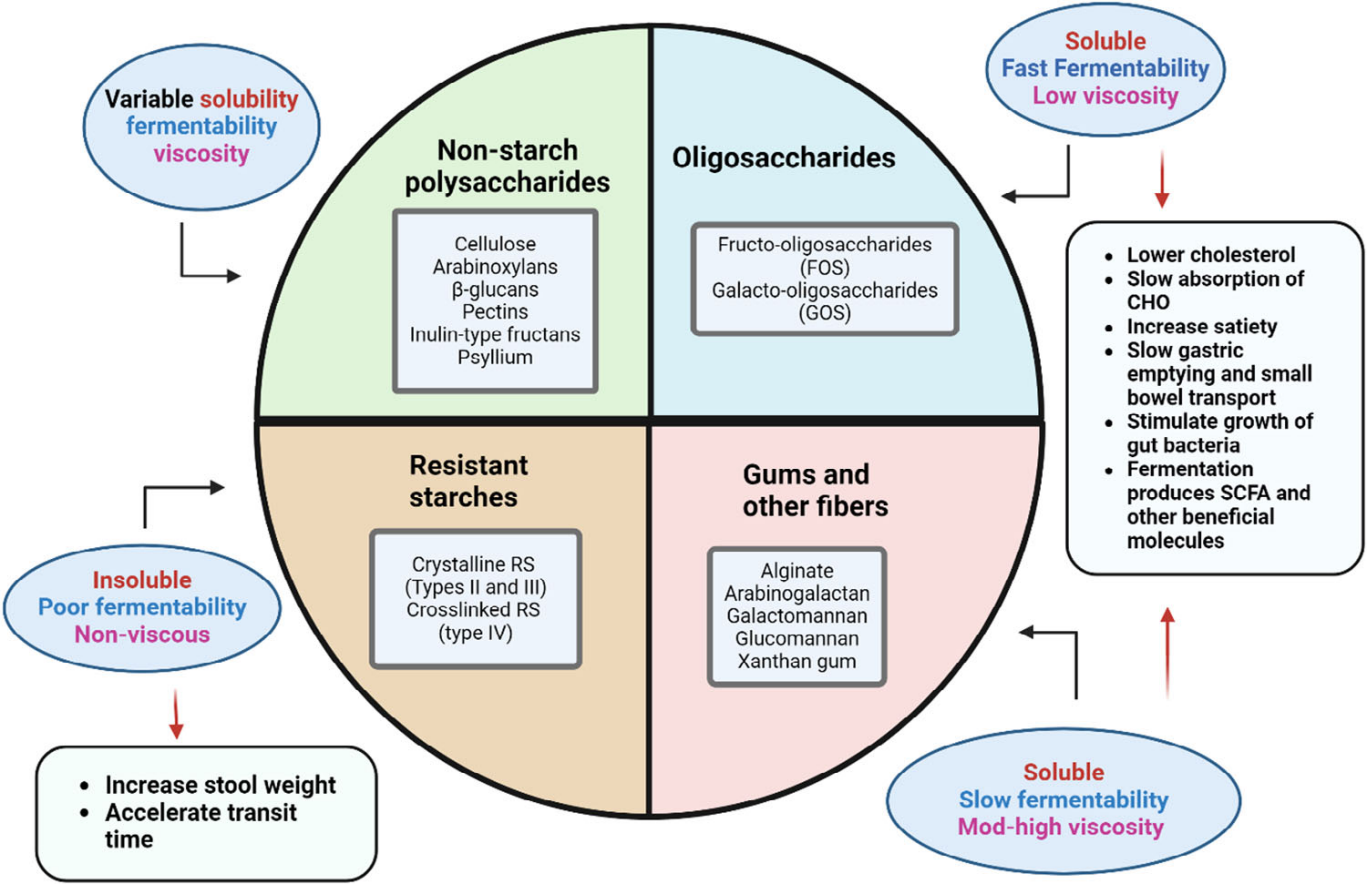
Classification of dietary fibres and physiological effects. Fibres are categorised based on physiochemical properties including solubility, viscosity, and fermentability. A large degree of heterogeneity exists between fibres regarding their physicochemical properties and subsequent physiochemical effects in the body. An understanding of these properties of isolated fibres is necessary in order to effectively design personalised therapies for treatment of specific metabolic disorders. CHO: carbohydrates. RS: resistant starch; SCFA: short‐chain fatty acids. Created with BioRender.com.

The greatest challenge of adopting fibre as a routine daily therapy for either prevention or treatment of disease includes the complex classifications of dietary fibres which can be subdivided by their physicochemical properties (solubility, viscosity and fermentability) (Figure 1). These physiochemical properties and the amount of fibre consumed determine how gastrointestinal function will be affected and what effects will be seen on host inflammation, metabolism, and energy homeostasis.^1^ The role of the gut microbiota also needs to be considered as fermentability of fibre by specific microbes produces a great number of molecules that are involved in modulation of metabolism. It is possible that benefits of fibre intake could be maximised in an individual by matching specific fibre consumption with existing microbial functional characteristics in the individual. The successful development of tools such as a stool test to examine the microbes found in each individual's gut will, in the future, help predict which fibres the individual could consume for the most benefit and least detrimental effects.

However, at the present time, the clinician is faced with an impossible task in which there is rapidly mounting evidence for dietary fibres advancing metabolic health, but little practical options for healthcare providers other than to simply recommend patients consume more fibre. If dietary fibres could be demonstrated to act as successful adjuncts to sustain or improve standard of care therapies or even alleviate common gastrointestinal side effects associated with current treatments, they would be an invaluable tool in our armamentarium. Unfortunately, currently the only method to determine individualised responses to fibre supplements is a ‘try it and see what happens’ approach.

## 2 SUMMARY

**FIGURE 2:**
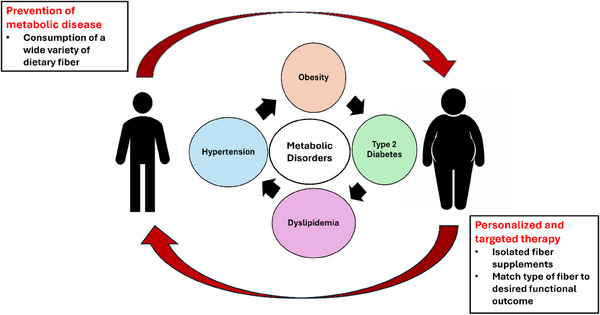
Use of fibre in the prevention and therapy of metabolic diseases. A diet encompassing a wide variety of foods high in fibre is recommended for prevention of disease. A more targeted approach may be beneficial in the treatment of metabolic disease using specific fibre combinations that are personalised to the individual and their specific gut microbes.

In conclusion, taken together, overwhelming evidence exists supporting the use of dietary fibre as a public health tool with which to prevent obesity and its metabolic consequences (Figure 2). There is also a large body of evidence supporting the use of fibre intake as adjunctive therapy for patients with hypertension, dyslipidaemia and T2DM and patients with these conditions should be encouraged to consume increased amounts of a variety of high fibre foods, including fruits, vegetables, grains, nuts and seeds, and legumes as well as specific fibre supplements.

## AUTHOR CONTRIBUTIONS

VM and KM contributed equally to the writing of this article.

## ETHICS STATEMENT

The authors have no competing interest to declare.
